# Lectures on Diseases of the Eye

**Published:** 1850-12

**Authors:** 


### LECTURES ON DISEASES OF THE EYE.

By reference to our advertising page, it will be seen that a course of lectures on the Anatomy, Physiology, and Diseases of the Eye will be given by Drs. Parrish and Neill, two of the surgeons of the Wills' Hospital for diseases of the eyes and limbs.

The institution in which it is proposed to deliver the above named lectures, is easy of access, contains about forty beds, and has a large dispensary attached to it, in which every variety of disease may be studied. The instruction will be of a thoroughly practical character, members of the class, which is limited to twenty-five, seeing for themselves the various operations and modes of treatment as well as their results, a mode of instruction which cannot be conveyed either by pictures or models, or in the amphitheatre.

We cannot too strongly recommend this matter to those of our readers who may be desirous of studying this too much neglected department. The gentlemen who have undertaken it are eminently qualified for the duty, and from the limited size of the class can have no other motive than that of advancing the interests of their pupils.

